# Eating a healthy lunch improves serum alanine aminotransferase activity

**DOI:** 10.1186/1476-511X-12-134

**Published:** 2013-09-14

**Authors:** Masako Iwamoto, Kaori Yagi, Kayoko Yazumi, Airi Komine, Bungo Shirouchi, Masao Sato

**Affiliations:** 1 Graduate School of Health and Nutrition Sciences, Nakamura Gakuen University, 5-7-1 Befu, Jonan-ku, Fukuoka, 814-0198, Japan; 2 Department of Nutritional Sciences, Faculty of Nutritional Sciences, Nakamura Gakuen University, 5-7-1 Befu, Jonan-ku, Fukuoka, 814-0198, Japan; 3 Laboratory of Nutrition Chemistry, Department of Bioscience and Biotechnology, Faculty of Agriculture, Graduate School of Kyushu University, 6-10-1 Hakozaki, Higashi-ku, Fukuoka, 812-8581, Japan

**Keywords:** ‘Once-a-day’, Dietary intervention, Serum alanine aminotransferase (ALT), Non-alcoholic fatty liver, Lunch, Cafeteria

## Abstract

**Background:**

Nutritional guidance and diet control play important roles in the treatment of obesity and non-alcoholic fatty liver. However, in Japan, nutritional guidance is difficult to provide in practice. Therefore, we evaluated the effects of providing the ‘once-a-day’ intervention of a healthy lunch on various metabolic parameters.

**Methods:**

For a 1-month preparatory period, 10 subjects generally consumed the lunches that were provided by the worksite cafeteria. This was followed by a 1-week washout period, after which, the subjects consumed healthy, low-calorie, well-balanced lunches for a 1-month test period. After the preparatory and test periods, blood samples were obtained from all subjects. The serum levels of indices relevant to metabolic syndrome and fatty liver were measured.

**Results:**

Serum alanine aminotransferase activity significantly decreased by 20.3% after the healthy intervention. However, the indices of metabolic syndrome did not significantly change. Analysis of the relationship between serum alanine aminotransferase activity and nutrient content indicated that the improvement of serum alanine aminotransferase status was due to the higher vegetable content and lower animal-source protein of the meals provided.

**Conclusions:**

In summary, the ‘once-a-day’ intervention of providing a healthy lunch improved serum alanine aminotransferase status. A diet high in vegetables and low in animal-based protein is important in maintaining a healthy condition.

## Introduction

In general, non-alcoholic fatty liver develops in concurrence with obesity. Kojima et al. [1] reported a twofold increase in the onset of non-alcoholic fatty liver over a period of 12 years (1989–2000) in 23,819 Japanese male subjects [2]. However, the prevalence of fatty liver significantly increased with increasing grades of obesity. The prevalence rates of fatty liver in non-obese, pre-obese (body mass index [BMI] less than 30 kg/m^2^), and obese subjects (BMI over 30 kg/m^2^) were 12.8%, 51.4%, and 80.4%, respectively [1]. The serum indicators of fatty liver are aspartate aminotransferase (AST), alanine aminotransferase (ALT), and gamma-glutamyl transpeptidase (γ-GTP) activities; the onset of fatty liver disease is generally accompanied by an increase in the levels of these indicators. Prevention and treatment of fatty liver consists of dietary controls and changes to exercise habits. However, obesity is a relatively minor concern for Japanese workers because fatty liver does not produce any subjective symptoms. Thus, they may not choose to follow nutritionist-recommended diets and may tend to neglect efforts to prevent and treat these diseases. In a Japanese study of diabetic care among subjects with type 2 diabetes mellitus, the dropout rate was 42.5% [3]. It has been difficult for outpatients to continue their self-care at home and in the workplace. Lowe *et al*. [4] reported an intervention study targeting energy and nutrient intake for a 3–4 month period in worksite cafeterias located in the USA. The participants were assigned to 1 of 2 environmental change conditions. One group was given low-energy-density foods, and nutrition labels were provided for lunch items. The second group was given the same environmental change as group 1, plus pricing incentives for purchasing low-energy-density foods and they were also further educated about low-energy-density foods. In that study, total energy and the ratio of energy from fat decreased, while the ratio of carbohydrates increased after the intervention periods of both conditions. However, this report did not determine any serum parameters for fatty liver. Therefore, we have developed a convenient method for intervening in the diets and lifestyles of Japanese workers.

We investigated the effects of a ‘once-a-day’ dietary intervention on markers of fatty liver and obesity in Japanese subjects. The dietary intervention consisted of providing healthy lunches in a worksite cafeteria. The study was conducted over a 2-month period, which included a 1-month observation period and a 1-month intervention period in pre-obese subjects. Moreover, we investigated which nutrients in the lunches had the greatest effects on improving these markers.

## Methods

### Study design

This was a study of dietary intervention. Firstly, to enable monitoring of the baseline daily nutrient intake, the subjects were asked to generally consume meals from the set lunch menu provided by the worksite cafeteria for a 1-month preparatory period. This was followed by a 1-week washout period. After the washout period, the subjects consumed low-calorie, well-balanced healthy lunches for a 1-month test period. The nutrient compositions of the lunches during the preparatory and test periods were calculated using Excel Eiyou-kun ver. 5.0 (Kenpaku-sha, Tokyo, Japan). The subjects’ daily nutrient intake was monitored for 3 days during the preparatory and test periods by means of dietary records for breakfasts and suppers.

### Subjects

Study subjects were recruited from among male employees of a company in Fukuoka, Japan. Fourteen men with hypertension and/or dyslipidaemia participated in this study. Four subjects dropped out during the study period, leaving a final sample of 10 men. Criteria for participation in this study included no history of smoking or use of prescribed medicines. The characteristics of the subjects at the beginning of the study are shown in Table 1. This study was approved by the Nakamura Gakuen University Committee (No. 11–002), in accordance with the Declaration of Helsinki. Written informed consent was obtained from all participants.

**Table 1:** Characteristics of the subjects

**Characteristics**	**Initial value**	**Minimum - Maximum**
*N*	10	
Age (years)	46.8±2.2	31 - 56
Height (cm)	172±2	162.0 - 177.2
Body weight (kg)	70.7±2.9	58.7 - 83.5
BMI (kg/cm^2^)	23.9±0.8	20.6 - 28.2
Abdominal circumference (cm)	87.8±2.1	79.7 - 96.6
Blood pressure		
SBP (mmHg)	124±4	104 - 150
DBP (mmHg)	75.6±3.0	60 - 92
Glucose (mg/dL)	90.6±1.7	82 - 98
ALT (U/L)	27.0±2.9	10 - 39
AST (U/L)	23.2±1.6	15 - 31
γ-GTP (U/L)	34.8±5.6	16 - 71
Total cholesterol (mg/dL)	219±11	170 - 260
HDL cholesterol (mg/dL)	53.9±3.6	39 - 81
LDL cholesterol (mg/dL)	137±10	80 - 180
Triacylglycerol (mg/dL)	157±20	56 - 248
NEFA (μEq/L)	525±62	266 - 914
HMW-adiponectin (μg/mL)	2.31±0.22	1.63 - 3.97

Data show mean±S.E.

Abbreviations: *BMI* Body mass index, *SBP* Systolic blood pressure, *DBP* Diastolic blood pressure, *ALT* Alanine aminotransferase, *AST* Aspartate aminotransferase, *γ-GTP* Gamma-glutamyltranspeptidase, *NEFA* Non-esterified fatty acid, *HMW-adiponectin* High molecular weight-adiponectin.

### Blood sampling and biochemical measurements

After the preparatory and test periods, blood samples were obtained from all subjects after an overnight fast. Blood samples were assayed for total cholesterol, high-density lipoprotein (HDL) cholesterol, low-density lipoprotein (LDL) cholesterol, triacylglycerol (TAG), non-esterified fatty acid (NEFA), glucose, AST, ALT, γ-GTP, and high-molecular-weight (HMW) adiponectin levels in a commercial laboratory (SRL, Fukuoka, Japan).

### Statistical analyses

Data on daily nutrient intake are expressed as mean ± standard deviation (SD). Clinical data are expressed as mean ± standard error (SE) (*n* = 10). The statistical differences between the end-point of the preparatory period and the end-point of the test period were determined using a two-tailed, paired *t*-test. The Pearson’s correlation coefficient analysis was used to assess the correlation between serum ALT activity and the nutrients of lunches, using the combined data from the preparatory and test periods (*n* = 20). Differences were considered significant at *P* < 0.05 for all statistical tests.

## Results

Four of the 14 subjects withdrew from the study, mainly because of failure to participate in the blood-collection protocol. Therefore, data from 10 subjects were available for analysis. Furthermore, body weight and blood pressure data were available for 9 of the 10 subjects and waist circumference data, for 8 subjects.

The baseline characteristics are shown in Table 1. The mean age was 46.8 ± 2.2 years (range, 31–56 years). The mean BMI was 23.9 ± 0.8 kg/m^2^ (range, 20.6–28.2 kg/m^2^).

Table 2 shows the nutritional value of the lunches during each period. The intervention lunches had significantly reduced levels of energy, protein, fat, and carbohydrate. In particular, the sources of protein in the lunches differed between the preparation and test periods. In the healthy lunches, animal-derived protein levels were significantly decreased, while plant-derived protein levels were increased. The fat-to-energy ratio was also significantly reduced, while protein and carbohydrate energy ratios were significantly increased. Levels of potassium, vitamin B_6_, folic acid, water-soluble dietary fibre, insoluble dietary fibre, and total fibre were increased. Levels of retinol equivalents, α-tocopherol, vitamin B_2_, saturated fatty acids (SFA), mono-unsaturated fatty acids (MUFA), and polyunsaturated fatty acids (PUFA) levels in the test period lunches decreased.

**Table 2:** Nutrients comparison of dietary intakes in lunches on 4-weeks of preparation period and test period

**Nutrients/lunch**	**Preparation period**	**Test period**	**Change**	***P *value**
*N*	10	10		
Energy (kcal)	713±43	594±34	-118	0.000
Total protein (g)	28.1±2.7	25.7±1.1	-2.4	0.017
Animal protein (g)	16.6±3.0	12.4±1.4	-4.2	0.002
Plant protein (g)	11.5±0.8	13.3±0.7	1.8	0.000
Fat (g)	25.1±2.9	16.7±1.8	-8.5	0.000
Carbohydrate (g)	89.8±3.7	85.6±3.8	-4.2	0.022
Percentage of energy				
Protein (%)	16.1±1.4	17.4±0.7	1.3	0.011
Fat (%)	29.9±1.4	24.9±1.4	-5.0	0.000
Carbohydrate (%)	54.0±1.7	57.7±1.1	3.6	0.001
Potassium (mg)	998±82	1092±53	94	0.016
Magnesium (mg)	100±8	102±5	2	0.375
Phosphorus (mg)	377±34	361±12	-16	0.158
Cryptoxanthin (μg)	17.9±9.5	12.3±1.7	-5.6	0.093
Retinol equivalents (μg)	207±48	167±19	-41	0.029
Vitamin D (μg)	2.95±1.26	4.23±1.27	1.28	0.061
α-Tocopherol (mg)	3.15±0.78	1.81±0.18	-1.34	0.000
Vitamin K (μg)	107±11	114±10	7	0.090
Vitamin B_1_ (mg)	0.374±0.095	0.321±0.042	-0.053	0.144
Vitamin B_2_ (mg)	0.393±0.059	0.340±0.016	-0.053	0.033
Niacin (mg)	6.52±1.02	6.48±0.63	-0.04	0.913
Vitamin B_6_ (mg)	0.502±0.054	0.609±0.037	0.107	0.000
Vitamin B_12_ (μg)	2.37±0.85	2.01±0.86	-0.36	0.400
Folic acid (μg)	120±9	140±7	20	0.001
Pantothenic acid (mg)	2.11±0.24	2.02±0.08	-0.09	0.355
Vitamin C (mg)	36.6±3.7	37.7±2.4	1.1	0.528
SFA (g)	5.87±1.31	3.72±0.62	-2.15	0.001
MUFA (g)	9.50±1.30	5.99±0.84	-3.51	0.000
PUFA (g)	6.83±1.04	4.70±0.38	-2.13	0.000
Cholesterol (mg)	129±49	98.4±14.8	-30	0.149
Water-soluble dietary				
fibre (g)	1.33±0.82	5.13±1.27	3.80	0.000
Insoluble dietary fibre (g)	3.73±0.28	4.67±0.13	0.94	0.000
Total dietary fibre (g)	6.02±0.96	10.4±1.3	4.39	0.000
Sodium (mg)	1523±167	1509±65	-14	0.818
Sodium chloride equivalent (g)	3.87±0.43	3.83±0.17	-0.04	0.789

Data show mean ± S.D.

*P* value: two-tailed paired *t*-test.

Abbreviations: *SFA* Saturated fatty acids: *MUFA*, Monounsaturated fatty acids, *PUFA* Polyunsaturated fatty acids.

The subjects’ serum profiles and measures of physical parameters after each period are shown in Table 3. Body weight; BMI; systolic and diastolic blood pressure; abdominal circumference; and serum level of glucose, AST, γ-GTP, total cholesterol, HDL cholesterol, LDL cholesterol, TAG, NEFA, and HMW adiponectin did not change significantly after the test period compared with the preparatory period (Table 3). Serum ALT activity, an index of liver function, was the only parameter that significantly decreased (Table 3).

**Table 3:** Comparison of clinical data after 4-weeks intake of preparation period and test period

**Characteristics**	**Preparation period**	**Test period**	**Change**	***P *value**
*N*	10			10
Body weight (kg)*	70.4±2.9	69.7±2.5	-0.1	0.551
BMI (kg/cm^2^)*	23.9±0.8	23.6±0.7	0.0	0.597
Abdominal circumference (cm)**	85.9±1.9	84.9±1.7	-0.5	0.503
Blood pressure*				
SBP (mmHg)	120±5	121±4	1	0.635
DBP (mmHg)	75.3±4.5	77.9±2.7	2.9	0.276
Glucose (mg/dL)	91.2±3.3	95.3±3.5	4.1	0.361
ALT (U/L)	26.2±3.8	19.6±1.9	-6.6	0.023
AST (U/L)	23.0±2.0	20.3±1.2	-2.7	0.066
γ-GTP (U/L)	36.7±6.5	36.0±7.9	-0.7	0.806
Total cholesterol (mg/dL)	204±8	212±8	7	0.216
HDL-cholesterol (mg/dL)	53.6±4.3	56.0±3.2	2.4	0.312
LDL-cholesterol (mg/dL)	124±8	130±8	7	0.316
Triacylglycerol (mg/dL)	152±31	138±20	-14	0.645
NEFA (μEq/L)	436±74	515±63	79	0.446
HMW-adiponectin (μg/mL)	2.66±0.19	2.97±0.20	0.31	0.124

Data show mean ± S.E. *n = 9, **n = 8.

*P* value: two-tailed paired *t*-test.

Abbreviations: *BMI* Body mass index, *SBP* Systolic blood pressure, *DBP* Diastolic blood pressure, *ALT* Alanine aminotransferase, *AST* Aspartate aminotransferase, *γ-GTP* Gamma-glutamyltranspeptidase* NEFA* Non-esterified fatty acid, *HMW-adiponectin* High molecular weight-adiponectin.

We calculated the Pearson's correlation coefficient between serum ALT activity and the nutrients in the lunches at the end-point (Table 4). The 4 nutrients of total protein, animal-source protein, vitamin B_2_, and SFA were positively correlated to serum ALT activity. The 5 nutrients of plant-source protein, carbohydrate energy ratio, folic acid, insoluble dietary fibre, and total dietary fibre were negatively correlated with serum ALT activity. In particular, increases in animal protein intake strongly correlated with increases of serum ALT activity. In addition, reduced plant-source protein intake and insoluble dietary fibre intake were associated with increased serum ALT activity.

**Table 4:** Correlations between ALT and nutrients of lunches in mixed data of a preparatory and test period (n=20)

**Nutrients/lunch**	**Correlation coefficient**	***P *value**
Energy (kcal)	0.3167	0.1864
Total protein (g)	0.6381	0.0033
Animal Protein (g)	0.6857	0.0008
Plant Protein (g)	-0.5304	0.0161
Fat (g)	0.3440	0.1493
Carbohydrate (g)	-0.1494	0.5417
Percentage of energy		
Protein (%)	0.3743	0.1144
Fat (%)	0.2503	0.3014
Carbohydrate (%)	-0.5106	0.0255
Potassium (mg)	-0.3394	0.1552
Retinol equivalents (μg)	-0.2165	0.3733
Vitamin D (μg)	0.2585	0.2853
α-Tocopherol (mg)	-0.0425	0.8630
Vitamin B_2_ (mg)	0.4864	0.0347
Vitamin B_6_ (mg)	0.0811	0.7414
Folic acid (μg)	-0.6354	0.0035
SFA (g)	0.4684	0.0431
MUFA (g)	0.3600	0.1300
PUFA (g)	0.0196	0.9364
Water-soluble dietary fibre (g)	-0.3681	0.1210
Insoluble dietary fibre (g)	-0.6696	0.0017
Total dietary fibre (g)	-0.4692	0.0427

Abbreviations: *SFA* Saturated fatty acids, *MUFA* Monounsaturated fatty acids, *PUFA* Polyunsaturated fatty acids.

## Discussion

We investigated whether serum parameters associated with metabolic syndrome and fatty liver were affected by a ‘once-a-day’ dietary intervention of eating a healthy lunch for 1 month. Serum ALT activity was significantly reduced, and serum AST activity tended to be reduced by the intervention, but the other parameters measured were not significantly affected. Serum ALT, AST, and γ-GTP activities are markers of liver function [1]. In patients with fatty liver, serum ALT, AST, and γ-GTP activities are increased. A notable sign of fatty liver is an increase in serum ALT activity without increases in AST or γ-GTP activity [5]. Resolution of fatty liver can lead to an amelioration of obesity [6]. On the other hand, Kojima *et al*. [1] have reported that non-obese subjects (BMI < 25 kg/m^2^) accounted for about half of all subjects with fatty liver. In this study, the general obesity parameters of body weight, BMI, and abdominal circumference did not change significantly. Therefore, the increase in the number of patients with fatty liver in Japan cannot be explained by the changes in these parameters. As in our study, when the relationship between the amelioration of fatty liver and obesity is to be investigated, measures of visceral fat area determined by computed tomography (CT) and body fat percentage may be needed [1]. Haufe *et al*. [7] reported that serum ALT activities decrease with decreasing visceral adipose tissue areas, identified on CT. Our observation of the amelioration of serum ALT activity in subjects undergoing a ‘once-a-day’ dietary intervention may be of great importance in nutritional guidance and intervention studies.

We determined the nutrients that were related to the change in serum ALT activity, so that these findings could be used for nutritional guidance; we attempted to identify the nutrients in the test-period lunches that caused reductions in serum ALT activity. In Table 2, the nutrients are classified into 3 types, depending on whether they significantly changed and the direction of the change (increase or decrease in content). The first category was for those nutrients that were significantly increased in the test-period lunches as compared to the lunches consumed during the preparation period. The second category was for nutrients that significantly decreased in the test period. The third category was for nutrients that did not significantly change from the preparatory period to the test period. In Table 4, the nutrients that significantly increased or decreased in Table 2 were analysed for a possible relationship with serum ALT activity. The carbohydrate-to-energy ratio, folic acid level, and insoluble and total dietary fibre content were negatively correlated to serum ALT activity, i.e., the lunches with more of these nutrients had a greater reduction in serum ALT activity. Thoma *et al*. [8] systematically reviewed relationships between lifestyle interventions and non-alcoholic fatty liver disease in adults. They summarized 5 studies of diet-only interventions that reported that glucose control improved the status of fatty liver. Positive correlations were found between ALT activity and the amount of protein, vitamin B_2_, and SFA in the lunches; these nutrients are therefore associated with the elevation of serum ALT activity (Table 4). We recognized that there were significant differences between the preparation and test periods on energy and major nutrients as energy sources except protein. However, between energy and these nutrient consumptions and serum ALT activity, there was no significant correlation. Therefore, we considered that “quality” of dietary consumption is more important than “quantity” of that. Moreover, we analysed whether nutrients that were negatively or positively correlated to serum ALT activity were derived from any particular source. The correlation coefficient for the relationship between folic acid and insoluble dietary fibre in the lunches was 0.9056 (*P* <0.001) and that for the relationship between folic acid and total dietary fibre was 0.7249 (*P* <0.001). As expected, the correlation coefficient for the relationship between total dietary fibre and insoluble dietary fibre in the lunches was high (0.8840; *P* < 0.001). The major foods groups containing folic acid and dietary fibre are vegetables and fruits [9]. These results suggest that a higher intake of vegetables and fruits, which have high levels of folic acid and insoluble dietary fibres, improves serum ALT activity. Qin’s intervention study was performed with 455 middle-aged Chinese men and showed that 0.8 mg of folic acid per day may contribute to lowering serum ALT activity [10]. Furthermore, with regard to the nutrients that were positively correlated with serum ALT activity, the correlation coefficient for the relationship between total protein and SFA in the lunches was 0.7880 (*P* < 0.001), between total protein and vitamin B_2_ was 0.8824 (*P* < 0.001), and between SFA and vitamin B_2_ was 0.7827 (*P* < 0.001). Moreover, the correlation coefficient for the relationship between animal protein and SFA in the lunches was 0.8241 (*P* < 0.001) and between animal protein and vitamin B_2_ was 0.8314 (*P* < 0.001). These nutrients are derived from animal-source foods [9]. There are no clinical data available from Japan on the relationship between serum ALT activity and the intake of animal-source foods. The results of this study suggest that reducing animal-source protein and increasing plant-source protein in the diet can improve hepatic function, as determined by beneficial changes in serum ALT activity. In Italian obese patients with BMIs ranging from 30.9 to 73.7 kg/m^2^, Ricci *et al*. [11] investigated the possible relationships between serum indicators of fatty liver and inadequate dietary intake of nutrients and energy. They showed that intake of high protein (>70 g/day), especially animal protein, was a risk factor for ALT elevation (odds ratio, 4.06; 95% confidence interval: 1.19–11.38). They also showed that an excessive intake of SFA (mean ± SD, 22.7 ± 16.1 g/day) was not associated with an increased risk of ALT elevation. In a 6-month dietary intervention study, Elias *et al*. [12] reported that a reduction of energy caused a significant decrease in serum γ-GTP activity and the ALT activity tended to decrease. In addition, the ratio of SFA to total energy intake and cholesterol content were decreased. The reductions of SFA and cholesterol are related to the reduction of animal products in the diets. In this study, SFA intake was low in both the preparation period (20.6 ± 7.7 g/day) and the test period (15.1 ± 3.6 g/day). These data suggest that a reduction of SFA intake may decrease serum ALT activity, and/or that the results from highly obese subjects in Italy have no effect on serum ALT activity. However, Japanese subjects with slightly elevated BMI (23.9 ± 2.4 kg/m^2^) may be susceptible to changes in serum ALT activity, modulated by SFA intake. Unfortunately, vitamin B_2_ intake was not measured in Ricci’s study [11].

A better understanding of the relationship between serum ALT status and nutrient intake can be obtained by use of a multi-regression analysis [13]. However, we did not use that analysis in this study because of the small number of subjects. We plan to conduct a ‘once-a-day’ dietary intervention study on a larger scale future.

In summary, a ‘once-a-day’ dietary intervention of a healthy lunch for 1 month improves serum ALT status. Promoting the intake of vegetables and fruits and limiting the intake of animal-source foods may be responsible for this effect; this information will be useful in providing nutritional guidance.

## Abbreviations

ALT: Alanine aminotransferase; AST: Aspartate aminotransferase; BMI: Body mass index; γ-GTP: Gamma-glutamyl transpeptidase; HMW-adiponectin: High molecular weight adiponectin; MUFA: Mono-unsaturated fatty acids; NEFA: Non-esterified fatty acid; PUFA: Polyunsaturated fatty acids; SFA: Saturated fatty acids; TAG: Triacylglycerol.

## Competing interests

The authors of the manuscript declare no conflicts of interest.

## Authors’ contributions

MI was responsible for the study design and the finalization of the report; K. Yagi participated in the technical analysis and contributed to data interpretation; K. Yazumi contributed to data interpretation and participated in the overall design; AK provided technical analysis; BS contributed to data interpretation and drafted the report; MS contributed to data interpretation, drafted the report, and participated in the overall design. All authors read and approved the final manuscript.
